# Investigation on the Atomic Mechanism for Grain Refinement of Magnesium Alloys by Mg-Zr Master Alloy

**DOI:** 10.3390/ma15207063

**Published:** 2022-10-11

**Authors:** Aimin Zhang, Jing Li, Fenglin Li, Guang Su

**Affiliations:** 1 Department of Materials Science and Engineering, Henan Institute of Technology, Xinxiang 453000, China; 2 Department of Electrical Engineering and Automation, Henan Institute of Technology, Xinxiang 453000, China

**Keywords:** heterogeneous nucleation mechanism, magnesium alloy, two-dimensional atomic layer, electronic band structure

## Abstract

The valence electron structure, bond energy, and cohesive energy of Mg, Zr, and α-Mg containing Zr, and α-Zr containing Mg crystals were calculated using the empirical electron theory of solids and molecules (EET). The calculation results show that the bond and cohesive energies of Zr were much greater than those of Mg, so Zr particles could precipitate ahead of α-Mg in general magnesium alloy melts or insoluble Zr particles exist when the magnesium melt temperature is relatively low. The bond energy of α-Zr decreases with the increase in Mg content; therefore, at the end of the growth of Zr particles, the remaining Zr atoms in the melt exist in the form of Mg-Zr clusters. In order to reduce the surface energy of Zr particles, the outer surface of Zr particles tends to terminate with a Zr-Mg atomic layer, that is, a stable two-dimensional Zr-Mg atomic layer is formed first on the (0001) crystal surface of the outermost surface of Zr particles. Furthermore, on the basis of the calculated results, a complementary criterion to the edge-to-edge model of heterogeneous nucleation is also proposed. {ure and single Zr particles cannot become the heterogeneous nucleus of α-Mg, but when there is an atomic layer of two-dimensional Zr-Mg on its surface, the nucleation of particles can be activated. Mg atoms in the liquid phase preferentially attach to the Zr-Mg/Mg-Zr atomic layer on the surface of Zr particles to grow and form a stable ordered structure, which lastly transforms Zr particles into efficient heterogeneous cores.

## 1. Introduction

The effective refinement of a solidification structure of as-cast magnesium alloys can improve both the strength and plasticity of the alloy, and the fluidity of liquid metal during solidification, inhibit porosity, and reduce the tendency of hot cracking and other casting defects [1,2,3]. For magnesium alloys without Al, Zr-containing refiners need to be added to the alloy melt to effectively refine the as-cast microstructure and grain size [4,5]. Although Zr has been used in magnesium alloys for decades, most early research work focused on how to add Zr to Mg melts and the gain-refining process [6,7]. The refining mechanism of Zr on magnesium alloys without Al is only theoretical, such as the atomic structure matching degree; the atomic action mechanism of the nucleation initial process of Mg atoms attached to Zr particles is especially not yet clear [8]. However, the essential mechanism resulting in grain refinement can not only be explained from the viewpoint of the crystallographic relationships between nucleant and matrix [9,10]. Whether and how a similar composition to that of the primary phase can be preferentially formed to adhere to nucleant surface may be another major influential factor and should be well-considered.

Regarding the grain refinement mechanism of Zr in magnesium alloys, in the early days, Sauerwald believed that only the dissolution Zr could refine the grain, while undissolved Zr particles had nothing to do with grain refinement [11,12]. In the 1960s, Emley [13] proposed the “peritectic reaction” mechanism and believed that the zirconium-rich solid solution (Zr halo structure) was formed by peritectic reaction between Zr particles precipitated from magnesium melt and magnesium liquid; therefore, only Zr particles that precipitated at peritectic temperature could promote the nucleation of Mg melts. However, the peritectic reaction mechanism cannot explain why Zr with lower than peritectic composition coyld also refine the grain size of α-Mg, and the Zr halo structure also exists. Subsequently, Tamura [14] and Qian [15] found that undissolved Zr also plays an important role in grain refinement, and proposed a heterogeneous nucleation mechanism of Zr particles based on a similar crystal structure to that between Zr and Mg. However, lattice mismatch is not a necessary condition for heterogeneous nucleation, and the interaction between a heterogeneous nucleus and matrix atoms is the key factor to determine whether or not the heterogeneous nucleation can occur [16].

Microstructural analysis of the halo structure shows that the center of the halo is a pure Zr particle surrounded by Zr-Mg solid solution, and the Zr content of this halo gradually decreases along the radius of the halo [17]. It is obvious that the halo with double nucleation and supernucleation structures formed by Zr particles and dissolved Zr atoms are similar to those of the Al–5Ti–1B master alloy refined on pure aluminum [18], of which the refining mechanism cannot simply be explained by heterogeneous nucleation theory. Because the Al–Ti two-dimensional transitional layer formed preferentially between the substrate and liquid atoms greatly impacts the final refining effect [19], it is necessary to indepth study the microscopic mechanism of the formation of the Zr halo, and the interaction between the heterogeneous phase and matrix atoms in the process of the heterogeneous nucleation initiation of the Mg alloy refined by Zr.

## 2. Calculation Method

EET, established by Yu in 1978 [20], is based on Pauling’s valence bond theory and Hume–Rothery’s electron concentration theory. With the two main problems of multiple solutions and calculation accuracy being improved by using the equal probability principle [21] and the self-consistent bond length difference (SCBLD) method [22,23], respectively, after decades of continuous efforts, the developed EET is widely used in many fields such as solid solutions, compounds, mechanical properties, strengthening mechanisms and phase transformations [24,25,26,27,28,29]. In this paper, it is therefore possible to calculate the bond energy and cohesive energy for Mg, Zr, Mg–*x*%Zr, and Zr–*x*%Mg using EET.

**Figure 1:**
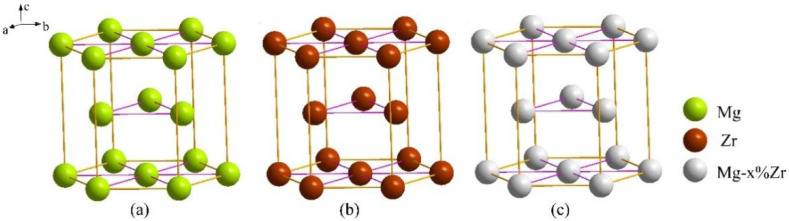
VES analysis models of (**a**) Mg, (**b**) Zr, (**c**) Mg-doped Zr (Mg–*x*Zr) structure unit.

The valence electron structure (VES) in EET usually includes covalent bonds formed by atoms, the electron distribution on covalent bonds, and the atomic states. Figure 1 shows the VES analysis model. Because Mg and Zr have the same crystal structure, three calculation models were adopted in this paper, i.e., the VES analysis model of Mg, Zr, and Mg-Zr/Zr-Mg, and their atomic arrangements in the unit cell are shown in Figure 1, respectively. The position of solution atom Mg in Zr or Zr in Mg is random and indeterminate. Therefore, according to the calculation model of the previous investigation, we consider that a kind of mixing atom that was composed of (1 − *x*)Mg/(1 − *x*)Zr and *x*Zr/*x*Mg atoms occupied the lattice point of the primary Mg/Zr, as shown in Figure 1c. There were four kinds of covalent bonds in the Mg and Zr structure unit, and the VES parameters for the four crystal structure could be calculated with the SCBLD method; the main equation and detail calculation steps for SCBLD method in EET are given in [22,23]. The corresponding calculation results of VES and cohesive energy are shown in Table 1. There were four kinds of covalent bonds in each analytical unit, and the meanings of CBN, EBL, $n_{\alpha }^{\prime }$, $E_{\alpha }^{\prime }$ parameters are also given in [23].

On the basis of the calculation of VES, the bond energies of covalent bonds and the cohesive energy of the four crystals can also be obtained. The main calculation equations of the bond energies ($E_{\alpha }^{\prime }$) in the structure unit are as follows:

(1) $$\begin{array}{l} E_{\alpha }^{\prime }=b\sum \frac{I\alpha n\alpha }{\bar{D}n\alpha }f\\ b=\frac{31.395}{n-0.36\delta }\\ f=\sqrt{\alpha s}+\sqrt{3\beta p}+g\sqrt{5\gamma d} \end{array}\}$$

where the meanings of the calculation parameters in Equation (1) are given in [23].

The calculation formula of cohesive energy ${\bar{E}}_{C}$ can be expressed as follows:

(2) $${\bar{E}}_{C}=b\left\{\sum_{\alpha }\frac{I_{\alpha }n_{\alpha }}{{\bar{D}}_{n\alpha }}f+\frac{n_{f}}{\bar{D}}f^{\prime }+km^{3d}-CW\right\}$$

where the meanings and solution of the above calculation parameters can also be found in [23].

In view of the multiple solutions of EET, the statistical value of cohesive energy of the four structure unites can be calculated according to [21,29,30]:

(3) $${\bar{E}}_{C}^{\prime }=\sum_{i=1}^{\sigma_{N}}{\bar{E}}_{Ci}\cdot c_{i}$$

where the meanings of the above calculation parameters are given in [23]. After the optimized lattice constants and *β* parameter had been obtained by using the SCBLD method, we could calculate the bond energies and cohesive energy of the four structure units on the basis of Equations (1)–(3). The partly calculated bond energies and cohesive energy are also listed in Table 1.

## 3. Results and Discussion

### 3.1. Precipitation of Zr Particles

**Figure 2:**
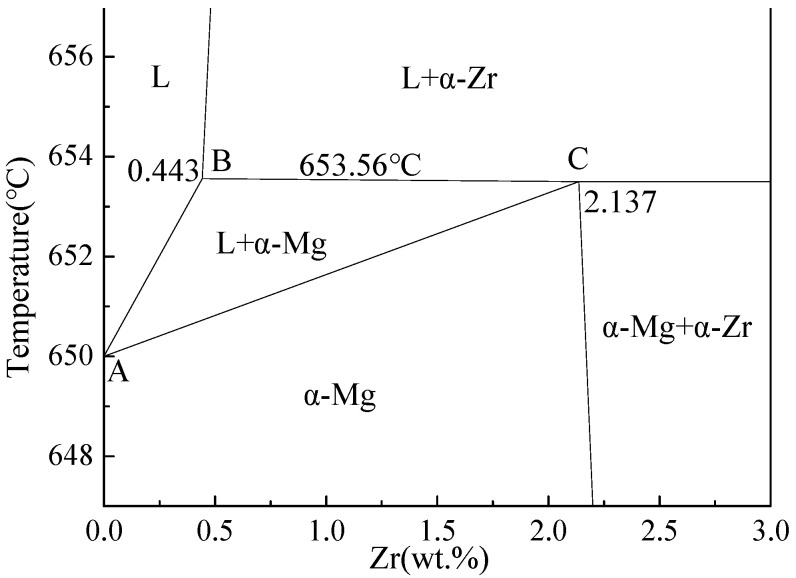
Peritectic corner of the Mg-Zr phase diagram [31].

The phase diagram [31] of the Mg-Zr binary alloy illustrated in Figure 2 shows that, when the temperature decreased to the peritectic reaction temperature, Zr particles precipitated from the Mg-Zr melt. As the Zr content increased from 0.443 to 2.137 wt.% of the peritectic component, the solidification time of the Mg-Zr alloys with different components was significantly shortened. At that time, a large number of Zr particles precipitated before the peritectic temperature, and their number also gradually increased with the increment of Zr content. In other words, the more Zr particles precipitated, the more conducive they were to the rapid solidification of the Mg-Zr melt and the formation of a fine solidification structure, from which we could infer that the first precipitated Zr could become an effective heterogeneous nucleus of α–Mg. Therefore, only from the phase diagram, we could confirm that the heterogeneous nucleation effect of Zr precipitated first.

In addition, the cohesive energy of Zr is much greater than that of Mg. From the calculation results of cohesive energy, it can also be inferred that, when the Zr content met the composition conditions, that is, the Zr content in Mg melt was greater than 0.45 wt.%, even above the peritectic temperature, Zr–Zr atomic clusters could quickly reach the critical nucleation size of transforming into Zr particles and then precipitated preferentially from the Mg-Zr melt. The literature [14,15] indicated that Zr particles that precipitated before peritectic reaction play a certain role in grain refinement. Because the solubility of Zr in Mg is stationary, the amount of precipitated Zr particles near the peritectic temperature was also correspondingly determined. So, when the content of Zr exceeded the peritectic content, the continuous increase in the refinement effect fully confirmed the grain refinement effect of Zr that precipitated first, of which the analytical result was the same as that of the phase diagram.

However, there were some different experimental phenomena from the phase diagram. When the Zr content exceeded a certain value, the refinement effect did not increase with the continuous increment of Zr content [32], which shows that not all Zr particles could become effective heterogeneous nuclei. Zr particles that precipitated first could be used as the nucleation core of the Zr following precipitation during peritection, and the amount of precipitated Zr during peritection is limited; hence, there is generally a surplus of first-precipitated Zr. Eventually, two different forms of Zr particles may be exist in a Mg-Zr melt, the first-precipitated Zr particles not surrounded by Zr atoms from peritectic reaction, which could be called “pure Zr”, and Zr particles attached to the solution Zr nucleated late during the peritectic reaction. It can be inferred from the literature that the first-precipitated Zr particles that are not surrounded by the peritectic precipitated Zr atoms may not play an important role in grain refinement. The main influence factor is obviously the difference of the two kinds of Zr particles. Therefore, it is necessary to investigate the surface composition and structure of the two kinds of precipitated Zr in detail.

### 3.2. Formation Mechanism of Two-Dimensional Zr-Mg/Mg-Zr Transitional Layer

When the Zr content in the melt reached the peritectic component point, the Zr dissolved in the Mg melt precipitated with the temperature decreasing to the peritectic temperature, and the whole melt then completed the peritectic reaction. The essence of the peritectic reaction is also a process of nucleation and growth. Through the continuous aggregation of Zr atoms in the melt, when the local Zr content reached a certain composition, fine Zr particles were precipitated to form the initial heterogeneous core. In fact, even when the Zr content was lower than the peritectic component, as the temperature decreased to the peritectic temperature, Zr atoms also gathered locally in the melt to form a stable structure similar to Zr particles. Therefore, Zr could create a certain refining effect when the Zr content was even lower than the peritectic component. However, under this condition, there were more Mg atoms in Zr-Mg clusters that hindered the aggregation of Zr atoms and the formation of Zr particles. The less the Zr content in Mg was, the more difficult the binding of Zr and the smaller the cohesive energy were. Regardless of the composition of Zr, the content of Mg in Zr-Mg clusters affected the cohesive energy of Zr during its precipitation, which significantly affected the precipitation process of Zr particles.

**Figure 3:**
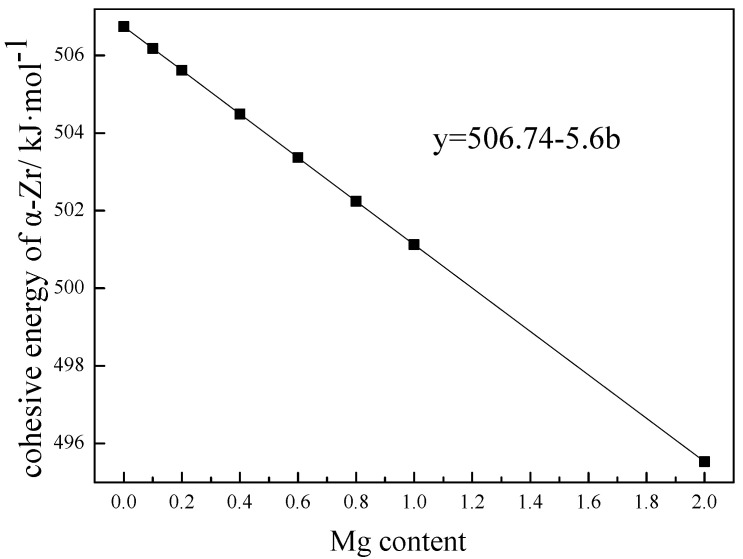
Cohesive energy of Zr with different Mg contents.

**Figure 4:**
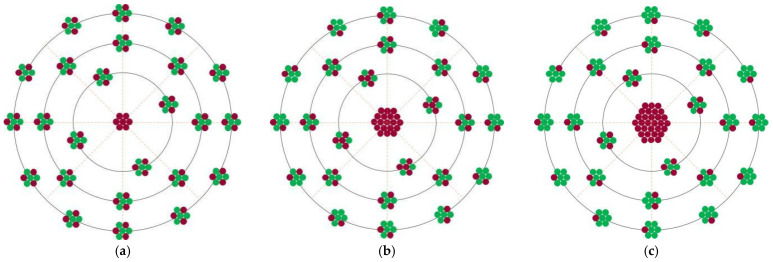
Schematic diagram of the formation of Zr-enriched halo structure in Mg-Zr alloy during solidification process (**a**) initial stage (**b**) intermediate stage (**c**) final stage.

Figure 3 shows the effect of Mg content on the cohesive energy of Zr-Mg solid solution. With the increase in Mg content, the cohesive energy of Zr-Mg obviously decreased proportionally. At the initial stage of solidification transformation, Zr-Mg clusters with less Mg content might have higher cohesive energy, and Zr was easy to diffuse and precipitates preferentially; the remaining Zr in Zr-Mg clusters with higher Mg content might have smaller cohesive energy, and Zr atoms prefer to adhere to the first-precipitated Zr particles to complete the precipitation transformation. When the temperature was low, the diffusion ability of Zr around the halo was poor. At that time, although the cohesive energy of Zr-Mg continued to decrease, the reduction in temperature provided a greater driving force for the phase transformation of the Zr-Mg cluster. At that time, Zr-Mg clusters were no longer attached to the precipitated Zr-Mg for growth, but could nucleate and precipitate independently. Therefore, the structure and morphology with a Zr -ich halo macroscopically formed [17], and the formation diagram is shown in Figure 4. The Zr content in different positions of the halo was obviously different. The Zr content in the center of the halo was the highest, which can be considered to be the pure Zr particles that precipitated first. When it was away from the center along the radius direction, the Zr content gradually decreased.

**Figure 5:**
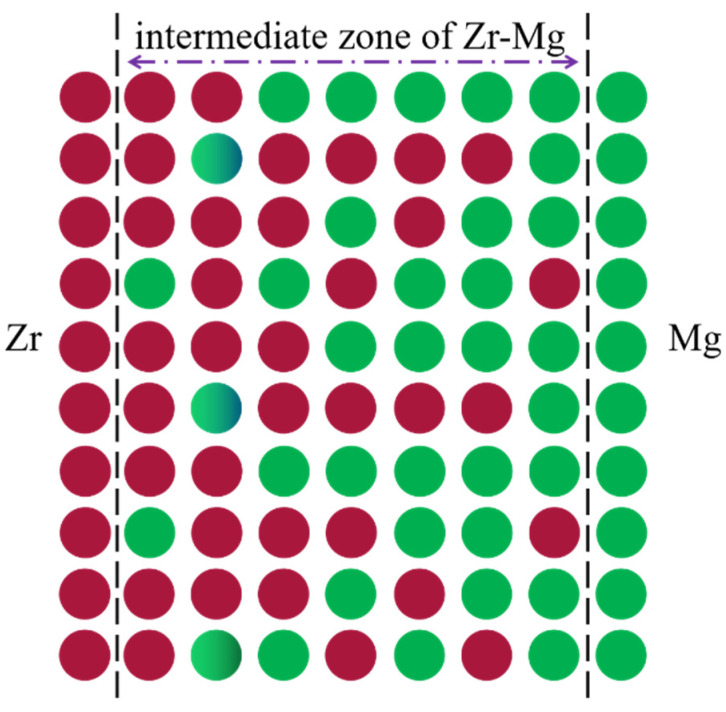
Schematic diagram of the formation of the Zr-Mg transitional layer on the surface of Zr particles during the solidification process.

Through the previous analysis, we can conclude that it was precisely because of the formation of this special halo structure in the precipitation process that some Zr particles can become an effective heterogeneous nucleus of α-Mg. In the microstructure, this structure of halo should be similar to the morphology of the double nucleation in an Al–Ti–B refiner, i.e., the two dimensional Al–Ti atomic layer was wrapped on the surface of TiB_2_ particles. Generally, the structure and composition of the outermost surface of such particles are the key factors to determine whether they can become a highly heterogeneous nucleus [16]. The outermost surface of the Zr-Mg halo structure must not be pure Zr atoms, but a solid solution transitional layer composed of Zr-Mg with different Mg contents. The structure of this transitional layer is still close to a hexagonal structure, and the Mg atoms in the liquid phase can be effectively attached to this transitional layer for growth at the initial stage of nucleation. The schematic diagram of the formation of the Zr-Mg transitional layer on first-precipitated Zr is shown in Figure 5. The Zr-Mg transitional layer has two main functions. On the one hand, the formation of a Zr-Mg transitional layer on the surface of Zr particles is the inevitable result of the reduction in surface energy in the later stage of Zr growth. The formation of a Zr-Mg surface with relatively low energy renders the whole system more stable. On the other hand, the formation of a Zr-Mg structure actually means that Mg atoms in the liquid phase completed partial nucleation with the condition of the melt temperature being slightly higher than that of the nucleation of α–Mg, which could lead to all Mg atoms remaining in liquid phase adhere to the stable Zr-Mg structure to continue heterogeneous nucleation and growth.

### 3.3. Atomic Mechanism of Heterogeneous Nucleation of Zr to α-Mg in Mg-Zr Alloy

There are two hypotheses about the refinement mechanism of Zr to Mg, heterogeneous nucleation and growth inhibition. Lee [33] indicated that, compared with other alloy elements in magnesium, dissolved Zr had the strongest growth inhibition effect on magnesium. However, an experimental study [34] showed that, after solid solution treatment, the grains of α-Mg can grow rapidly, which indicates that Zr has little inhibitory effect on the growth of α-Mg. In addition, no Zr element is found at the grain boundary of α-Mg. During the solid solution treatment, a small amount of Zr in the halo structure rapidly diffused to the nucleus; therefore, the grain refinement effect of Zr on Mg was not to inhibit grain growth, and the growth restrain factor (GRF) theory may not be applicable to peritectic elements.

**Figure 6:**
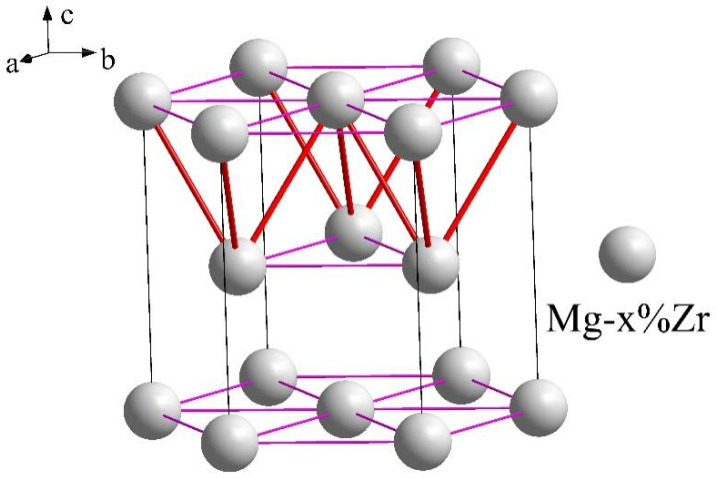
Primary bond distribution in the cell of Mg–*x*%Zr crystal structure; primary keys are marked with thick red lines.

On the basis of the above analysis and the research results in the literature, it can be inferred that the effect of adding Zr should be to promote the nucleation of α-Mg, of which the main function is heterogeneous nucleation. The essence of heterogeneous nucleation is that the surface of heterogeneous particles is strongly attracted to atoms in melt, which enables the atoms from the liquid phase to form a stable solid-phase structure on the surface of the heterogeneous phase, even when the melt temperature is slightly higher than its own crystallization temperature. Bond distribution in the Mg-Zr unit cell also shows that Zr atoms were strongly attracted to Mg atoms, as shown in Figure 6. Zr has the same crystal structure as Mg, and their lattice constants were close. The distribution of primary bonds of HCP structure, such as Mg/Zr, is shown in Figure 6. The atomic force between the (0001) crystal planes was the strongest. Therefore, the dissolution of Zr in Mg could increase the atomic interaction force between (0001) crystal planes on the Zr surface and in the Mg matrix, which could further increase the stability of Mg atoms attached to a heterogeneous nucleus. During the precipitation process, Zr atoms on the Zr-Mg surface layer firmly help in grasping Mg atoms in a liquid alloy. Even if a stable structure such as Zr particles cannot be formed, Mg-Zr clusters that are more stable than Mg–Mg clusters can also be formed, which can also greatly reduce the critical nucleation size of Mg–Mg clusters, thus promoting the subsequent nucleation of α-Mg.

**Figure 7:**
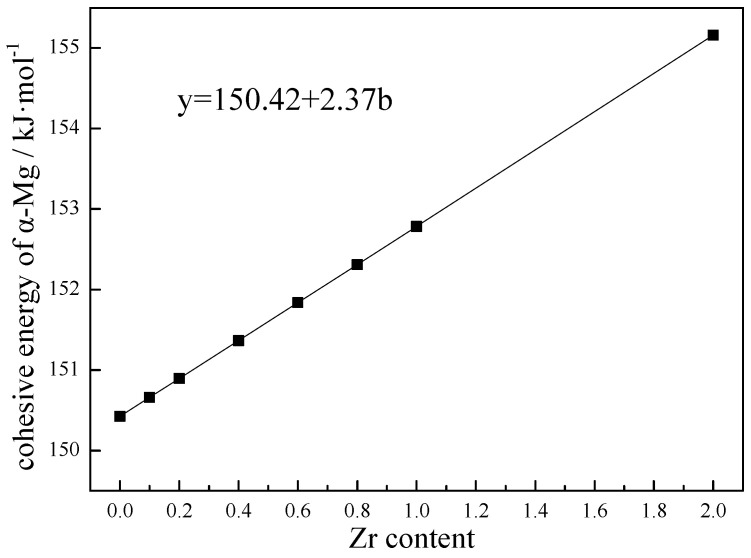
Cohesive energy of Mg with different Zr contents.

On the other hand, the incorporation of Zr into Mg increases the cohesive energy of Mg. As shown in Figure 7, the higher the cohesive energy is, the more preferential the formation of a solid Mg-Zr solution is. As shown in Figure 7, the cohesive energy of Mg-Zr increased with the increase in Zr content. That is to say, a solid Mg-Zr solution with large cohesive energy should be formed prior to that of pure Mg. In a word, a more stable two-dimensional Zr-Mg/Mg-Zr transitional layer formed on the surface of Zr particles can play an effective role in the heterogeneous nucleation of α-Mg.

**Figure 8:**
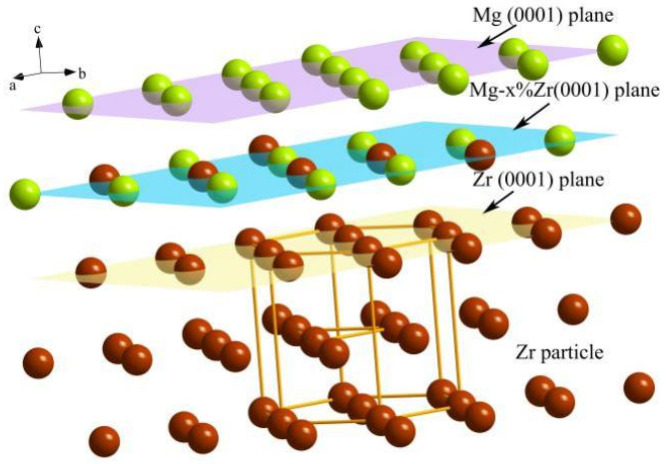
Schematic diagram of the atomic mechanism of heterogeneous nucleation in an Mg-Zr alloy.

Figure 8 shows the schematic diagram of the heterogeneous nucleation atomic mechanism in the Mg-Zr alloy. The two-dimensional transitional layer of Mg-Zr clearly played a crucial role in the heterogeneous nucleation process of Zr to Mg. The solid solution of Zr in Mg increased the interaction between Mg atoms, facilitating Mg–Mg clusters reaching the critical nucleation size. At the same time, it also reduced the transitional resistance of liquid-phase clusters to a solid phase. The required undercooling for nucleation was also reduced, so that the Mg–Mg atomic layer of the (0001) plane became more stable under the action of Zr. So, the nucleation and subsequent growth of Mg atoms in a liquid alloy could be completed at a higher temperature than the melting-point temperature of α-Mg, which is also the atomic essence of a peritectic reaction in an Mg-Zr system.

To sum up, the heterogeneous nucleation of Zr is the combination of Zr particles and dissolved Zr. Its atomic mechanism can be summarized as follows: Zr particles provide a stable hetero–structure similar to that of Mg, which meets the structural requirements of heterogeneous nucleation. A small amount of dissolved Zr in Mg forms a stable two-dimensional Zr-Mg/Mg-Zr transitional layer on the surface of Zr particles, so that some Mg atoms in the liquid phase can preferentially form a more stable solid phase, that is, the initial nucleation process of Mg atoms in liquid phase is completed. The efficient heterogeneous nucleus should meet the two conditions of an interface atomic structure and atomic composition.

## 4. Conclusions

The valence electron structure, bond energy, and cohesive energy of four different crystals, i.e., Mg, Zr, α-Mg containing Zr, and α-Zr containing Mg were calculated using the EET method. According to the calculation results and confirmed experimental results in previous work, we reached the following conclusions.

(1) The core-shell structure formed by dissolving Zr attached to the first-precipitated Zr particles is the initial core of heterogeneous nucleation, and the refining mechanism of Zr to Mg is heterogeneous nucleation.
(2) The dissolved Zr in the Mg-Zr melt formed a transitional layer of Zr-Mg/Mg-Zr on the surface of Zr particles. This two-dimensional transitional layer played a role in stabilizing the Mg atoms and completing the initial process of nucleation, which is key to the heterogeneous nucleation of α-Mg by Zr.
(3) The essence of heterogeneous nucleation is that atoms on the surface of heterogeneous particles strongly interact with atoms in the liquid phase, which should generally meet two requirements, i.e., a similar structure and composition between the heterogeneous and parent phases.

## Figures and Tables

**Table 1:** Calculated VES parameters, bond energies, and cohesive energies for Mg and Zr.

Crystals	CBN	EBN	EBL (nm)	$n_{\alpha }^{\prime }$	$E_{\alpha }^{\prime }$ (kJ/mol)
Mg	$B_{n1}^{\mathrm{Mg}-\mathrm{Mg}}$	6	0.31969	0.10998	10.2658
	$B_{n2}^{\mathrm{Mg}-\mathrm{Mg}}$	6	0.32094	0.10558	9.8168
	$B_{n3}^{\mathrm{Mg}-\mathrm{Mg}}$	6	0.45299	0.00142	0.0933
	$B_{n4}^{\mathrm{Mg}-\mathrm{Mg}}$	2	0.52103	0.000154	0.0088
${\bar{E}}_{C}=150.4251$					
Zr	$B_{n1}^{\mathrm{Zr}-\mathrm{Zr}}$	6	0.31788	0.26042	42.6535
	$B_{n2}^{\mathrm{Zr}-\mathrm{Zr}}$	6	0.32320	0.21849	35.1964
	$B_{n3}^{\mathrm{Zr}-\mathrm{Zr}}$	6	0.45333	0.00298	0.34244
	$B_{n4}^{\mathrm{Zr}-\mathrm{Zr}}$	2	0.51470	0.00039	0.03981
${\bar{E}}_{C}=506.7442$					

## Data Availability

Not applicable.
